# Association Between Respiratory Viral Infection and Peripheral T Lymphocyte Subsets in Elderly Patients With Acute Exacerbation of Bronchiectasis

**DOI:** 10.1111/crj.70151

**Published:** 2025-12-25

**Authors:** Guoping Zhang, Yuan Zhuang, Sai Wang, Qun Wang, Jun Qin, Zhi Yuan, Jing Zheng

**Affiliations:** ^1^ the People's Hospital of Fenghua Ningbo Ningbo Zhejiang Province China

**Keywords:** acute exacerbation, bronchiectasis, elderly patients, respiratory viral infection, T lymphocytes subsets

## Abstract

**Background:**

Respiratory viral infections are increasingly recognized as important triggers of acute exacerbations in bronchiectasis (AEB); the virological profiles and immunological mechanisms in elderly patients remain poorly characterized.

**Methods:**

A prospective cohort of 102 elderly bronchiectasis patients was followed for 12 months. Upon AEB occurrence, nasopharyngeal swabs were obtained for multiplex fluorescence quantitative PCR detection of eight respiratory viruses, while peripheral blood samples were analyzed for T lymphocyte subsets by flow cytometry. Clinical characteristics, inflammatory markers, and T cell subsets were compared between virus‐positive and virus‐negative groups at first AEB; ROC curve analysis evaluated the predictive value of T cell subsets for viral infection.

**Results:**

A total of 93 AEB episodes were captured from 68 patients during 12‐month follow‐up, with viruses detected in 54.8% (51 of 93) of episodes, influenza virus being the most common pathogen (24 of 51, 47.1%). Compared with the virus‐negative group, the virus‐positive group showed higher IL‐6 and TNF‐α and lower CRP and WBC levels (*p* < 0.05), higher sputum culture positivity with *Haemophilus influenzae* predominating, along with increased use of intravenous antibiotics and respiratory support, while quality of life, pulmonary function, and oxygenation remained comparable. The virus‐positive group showed lower CD4^+^ T cell counts (431.28 ± 152.36 vs. 508.42 ± 158.94, *p* = 0.025) and CD4^+^/CD8^+^ ratios (1.41 ± 0.44 vs. 1.63 ± 0.49, *p* = 0.013).

**Conclusions:**

Viral infections are frequent in elderly bronchiectasis patients with AEB and are characterized by reduced CD4^+^ T‐cell counts, lower CD4^+^/CD8^+^ ratios, and heightened inflammatory responses, reflecting underlying age‐related immune vulnerability.

## Graphical abstract

Respiratory viral infections are common in elderly patients with acute exacerbation of bronchiectasis. Viral‐positive episodes show distinct inflammatory profiles and reduced CD4^+^ T cells with lower CD4^+^/CD8^+^ ratios. The CD4^+^/CD8^+^ ratio demonstrates only moderate ability to indicate viral infection, suggesting limited value for independent clinical decision‐making.

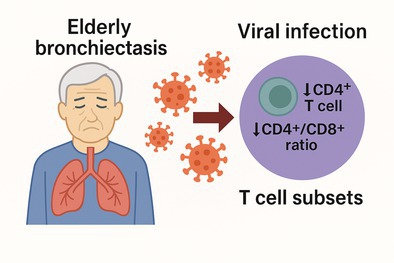

## 1 Introduction

Bronchiectasis is a chronic airway disease characterized by irreversible airway dilatation. Acute exacerbation of bronchiectasis (AEB) is a major cause of hospitalization, reduced quality of life, and disease progression, manifesting as worsening cough, increased sputum production with purulent changes, and dyspnea [1]. While bacterial infections have traditionally been considered the primary trigger of AEB, emerging evidence highlights the significant role of viral pathogens [2]. Prospective studies have reported viral detection rates of 25%–49% during AEB episodes, significantly higher than during stable periods, with rhinovirus and influenza virus being the most frequently identified pathogens [3, 4]. A 2024 multi‐omics study detected viruses in 46% of 120 AEB patients and confirmed significant disease heterogeneity [1].

T lymphocytes represent central effector components of the adaptive immune system and are critical for antiviral immunity: CD4^+^ T cells orchestrate immune responses through cytokine secretion, while CD8^+^ T cells directly eliminate virus‐infected cells [5]. In addition, the CD4^+^/CD8^+^ ratio, as a key indicator of immune homeostasis, has been associated with adverse clinical outcomes across multiple disease settings, including HIV infection, COVID‐19, and chronic viral hepatitis [6, 7, 8]. Evidence on the relationship between respiratory viral infection and T‐lymphocyte subset alterations during AEB in the elderly remains scarce. This study aimed to characterize viral infections in elderly AEB patients and assess their association with peripheral T lymphocyte subsets to advance understanding of the immunopathogenesis of AEB in this population.

## 2 Materials and Methods

### 2.1 Study Population

Patients with bronchiectasis who presented to the Department of Respiratory Medicine at Fenghua District People's Hospital of Ningbo between October 2024 and September 2025 were prospectively followed for 12 months. Inclusion criteria: (1) Age ≥ 70 years; (2) Meeting the diagnostic criteria of the 2021 “Expert Consensus on Diagnosis and Treatment of Bronchiectasis in Chinese Adults” [9], which is based on high‐resolution CT findings and clinical symptoms; (3) Stable condition with ability to comply with follow‐up; (4) Informed consent provided with signed informed consent form. Exclusion criteria: (1) Concurrent severe cardiac, hepatic, or renal dysfunction, active malignancy, or immunodeficiency; (2) Expected survival < 12 months; (3) Unable to comply with follow‐up or incomplete clinical data. The study protocol was approved by the Ethics Committee of Fenghua District People's Hospital of Ningbo (Approval No. 2024‐47‐K).

Diagnostic criteria for AEB included the worsening of at least three of the following symptoms lasting for ≥ 48 h: (1) cough; (2) sputum volume and/or consistency; (3) sputum purulence; (4) dyspnea and/or decreased exercise tolerance; (5) fatigue and/or malaise; (6) hemoptysis, with clinical judgment by physicians indicating a need for treatment regimen modification [10].

### 2.2 Study Procedure

Baseline data including demographics, medical history, lung function, and imaging were collected at enrollment when patients were in a stable state. Monthly follow‐ups (telephone or clinic‐based) monitored AEB occurrence. At each AEB episode requiring hospitalization, nasopharyngeal swabs for viral testing and blood samples for T cell subsets and inflammatory markers were collected within 24 h of admission. Repeated assessments were conducted for each episode of AEB.

Patients were divided into virus‐positive and virus‐negative groups based on viral detection results at the first time of AEB.

### 2.3 Respiratory Viral Pathogen Detection

Nasopharyngeal swabs were collected within 24 h of admission for AEB, stored in viral transport medium at −80°C, and tested within 48 h. Multiplex real‐time PCR detected 8 pathogens: influenza A (H1N1, H3N2), influenza B (Victoria, Yamagata), adenovirus, rhinovirus, parainfluenza 1–4, human metapneumovirus, coronavirus (229E, OC43, NL63, HKU1), and respiratory syncytial virus (RSV) A/B. Positivity was defined as Ct ≤ 35.

### 2.4 T Lymphocyte Subset Analysis

Fasting morning blood samples (2 mL) were collected in EDTA tubes within 24 h of admission. T lymphocyte subsets were analyzed by flow cytometry to determine absolute cell counts of CD3+, CD4+, and CD8^+^ populations, and the CD4^+^/CD8^+^ ratio was calculated.

### 2.5 Bacterial Culture

Sputum samples obtained during AEB were submitted for routine bacterial culture and pathogen identification using standard microbiological procedures.

### 2.6 Clinical Assessments

Baseline characteristics including demographics (age, sex, BMI, and smoking history), bronchiectasis duration, number of AEB episodes in the preceding 2 years, comorbidities. For each hospitalized AEB episode, the following data were systematically collected: (1) laboratory parameters including white blood cell count, neutrophil percentage, C‐reactive protein (CRP), interleukin‐6 (IL‐6), interleukin‐8 (IL‐8), and tumor necrosis factor‐α (TNF‐α); (2) sputum culture results; (3) spirometric indices including forced expiratory volume in 1 s (FEV_1_), forced vital capacity (FVC), and FEV_1_/FVC ratio; (4) partial pressure of oxygen (PaO_2_); (5) therapeutic interventions including antibiotic use, systemic corticosteroids, mucolytics, bronchodilators, and respiratory support; (6) length of hospital stay; (7) quality of life assessments using the St. George's Respiratory Questionnaire (SGRQ), Leicester Cough Questionnaire (LCQ), and COPD Assessment Test (CAT).

## 3 Statistical Analysis

Data analysis was performed using R software (version 4.4.3). Continuous variables were tested for normality and presented as mean ± standard deviation (x̄ ± SD) for normally distributed data, with between‐group comparisons conducted using independent samples *t*‐tests. Categorical variables were expressed as frequencies and percentages [*n* (%)], with between‐group comparisons performed using chi‐square tests or Fisher's exact test. ROC analysis evaluated predictive performance of indicators using AUC with 95% CI. *p* < 0.05 considered statistically significant.

## 4 Results

### 4.1 Viral Detection

Of the 102 enrolled bronchiectasis patients, 68 experienced at least one AEB during the 12‐month follow‐up period, with 93 AEB episodes recorded.

Respiratory viruses were detected in 51 of 93 AEB episodes (54.8%), comprising 45 single viral infections and 6 dual co‐infections (11.8% of virus‐positive episodes), with no triple or higher‐order co‐infections observed. A total of 45 patients experienced only 1 AEB episode, 21 patients experienced 2 AEB episodes, and 2 patients experienced 3 AEB episodes (Table 1). During the first AEB, 37 patients detected positive and 14 additional positive cases were identified in subsequent AEB episodes (Table 1). Only three patients exhibited the same viral pathogen across different episodes. Influenza virus was the most frequently detected pathogen in 24 cases (42.1%), followed by rhinovirus in 14 cases (24.6%), RSV in 10 cases (17.5%), and human metapneumovirus in five cases (8.8%). The most common dual infection combination was influenza virus plus rhinovirus (3 cases) (Table 2).

**TABLE 1:** Viral detection in AEB events.

Item	Total events (*n*)	Virus‐positive (*n*)	Single virus/dual viruses	Positive rate (%)
All AEB events	93	51	45/6	54.8
First AEB	68	37	33/4	54.4
Recurrent AEB	25	14	12/2	56.0

**TABLE 2:** Distribution of respiratory viruses in virus‐positive events.

Virus type	*n* (%)
Influenza virus	24 (42.1)
Rhinovirus	14 (24.6)
RSV	10 (17.5)
Human metapneumovirus	5 (8.8)
Others	4 (7.0)
Total	57 (100)

### 4.2 Baseline Characteristics

Among the 68 patients experiencing their first AEB in this study, 37 were virus‐positive and 31 were virus‐negative. Baseline characteristics of these patients, collected at enrollment during the stable state, are shown in Table 3. The two groups were comparable in demographic features (age, sex, BMI, and smoking history) and most disease characteristics (bronchiectasis duration, prior 2‐year AEB frequency, multiple lobes involved on HRCT, and number of comorbidities) (*p* > 0.05). By comparison, virus‐positive patients had higher Bronchiectasis Severity Index (BSI) scores and more frequent use of inhaled corticosteroids (*p* < 0.05) (Table 3).

**TABLE 3:** Comparison of baseline characteristics between virus‐positive and virus‐negative groups at stable state.

Variable	Virus‐positive group (*n* = 37)	Virus‐negative group (*n* = 31)	*t*/*χ* ^2^	*p*
Age (years)	77.26 ± 6.89	75.71 ± 5.91	1.218	0.226
Sex (Male/Female)	25 (67.6)	15 (48.4)	1.831	0.176
BMI (kg/m^2^)	21.18 ± 3.4	21.08 ± 3.05	0.154	0.878
Smoking history (yes)	30 (81.1)	22 (71.0)	0.479	0.489
Duration of bronchiectasis (years)	7.07 ± 6.98	6.39 ± 8.32	0.429	0.669
Prior 2‐year AEB frequency	2.67 ± 1.09	2.35 ± 0.98	1.290	0.202
Long‐term inhaled corticosteroids use (yes)	29 (78.4)	13 (41.9)	8.005	0.005
Multiple lobes involved on HRCT (yes)	24 (64.9)	24 (77.4)	0.747	0.387
Number of comorbidities				
0	8 (21.6)	10 (32.3)	1.015	0.602
1	20 (54.1)	15 (48.4)		
≥ 2	9 (24.3)	6 (19.4)		
BSI score	9.34 ± 2.99	7.65 ± 2.18	2.692	0.009

Abbreviations: BMI, body mass index; BSI, Bronchiectasis Severity Index; HRCT, high‐resolution computed tomography.

### 4.3 Impact of Viral Infection on Clinical Parameters

As detailed in Table 4, which presents data collected during AEB, there were differences in clinical presentation, laboratory findings, and treatment characteristics between virus‐positive and virus‐negative patients. Quality of life scores (SGRQ, LCQ, and CAT), lung function parameters (FEV_1_, FVC), and admission oxygenation indices were numerically lower in the virus‐positive group versus the virus‐negative group, without reaching statistical significance (*p* > 0.05). Regarding bacterial infection, the virus‐positive group demonstrated a significantly higher rate of positive sputum cultures (75.7% vs. 48.4%, *p* < 0.05), with *Haemophilus influenzae* being the predominant pathogen. Regarding inflammatory markers, virus‐positive patients had significantly higher IL‐6 and TNF‐α levels (*p* < 0.001) but lower CRP and white blood cell counts (*p* < 0.05), while IL‐8 levels were similar between the two groups (*p* > 0.05).

**TABLE 4:** Clinical and laboratory parameters during the first AEB in virus‐positive versus virus‐negative patients.

Variable	Virus‐positive group (*n* = 37)	Virus‐negative group (*n* = 31)	*t*/*χ*2	*p*
quality of life assessment				
SGRQ score	46.52 ± 10.82	49.09 ± 9.55	−1.040	0.302
LCQ score	12.95 ± 3.05	13.86 ± 2.82	1.275	0.207
CAT score	15.65 ± 5.55	16.35 ± 5.63	0.508	0.613
Pulmonary function and oxygenation				
FEV_1_ (L)	1.27 ± 0.34	1.41 ± 0.42	−1.437	0.156
FVC (L)	2.15 ± 0.65	2.27 ± 0.49	−0.846	0.401
PaO_2_/FiO_2_ ratio	271.38 ± 49.58	292.13 ± 38.08	−1.950	0.055
Bacterial culture				
Positive culture, *n* (%)	28 (75.7)	15 (48.4)	4.293	0.038
Inflammatory markers				
IL‐6 (pg/mL)	28.9 ± 7.88	18.23 ± 7.31	5.788	< 0.001
TNF‐α (pg/mL)	14.77 ± 3.93	10.98 ± 3.98	3.926	< 0.001
IL‐8 (pg/mL)	24.75 ± 8.83	22.12 ± 7.7	1.313	0.194
CRP (mg/L)	31.4 ± 19.07	46.88 ± 23.6	−2.937	0.005
WBC (×10^9^/L)	8.35 ± 2.69	10.2 ± 2.91	−2.700	0.009
Treatment strategy				
IV antibiotics, *n* (%)	34 (91.9)	20 (64.5)	3.386	0.007
Systemic steroids, *n* (%)	18 (48.6)	13 (41.9)	0.096	0.757
Mucolytics, *n* (%)	30 (81.1)	28 (90.3)	—	0.326
Bronchodilators, *n* (%)	30 (81.1)	24 (77.4)	0.005	0.944
Clinical outcomes				
Hospital stay (days)	9.03 ± 3.37	9.26 ± 2.73	−0.312	0.756
Respiratory support, *n* (%)	20 (54.1)	8 (25.8)	4.452	0.035

Abbreviations: CAT, COPD Assessment Test; CRP, C‐reactive protein; FEV_1_, forced expiratory volume in 1 s; FVC, forced vital capacity; IL, interleukin; IV, intravenous; LCQ, Leicester Cough Questionnaire; PaO_2_/FiO_2_, arterial oxygen partial pressure to fractional inspired oxygen ratio; SGRQ, St. George's Respiratory Questionnaire; TNF‐α, tumor necrosis factor‐alpha; WBC, white blood cell count.

Intravenous antibiotic use was more frequent in the virus‐positive group (*p* < 0.05), whereas systemic corticosteroids, mucolytics, and bronchodilator usage rates were similar between groups (*p* > 0.05). Furthermore, respiratory support requirements (noninvasive ventilation or high‐flow oxygen therapy) were more frequent in the virus‐positive group (*p* < 0.05). Hospital stay duration was similar between groups (*p* > 0.05).

### 4.4 Peripheral Blood T Lymphocyte Subset Changes

Peripheral blood T lymphocyte subsets were compared between virus‐positive and virus‐negative patients during their first AEB episode. The absolute CD4^+^ T‐cell count was significantly lower in the virus‐positive group (431.28 ± 152.36 vs. 508.42 ± 158.94, *p* = 0.025), along with a significantly reduced CD4^+^/CD8^+^ ratio (1.37 ± 0.48 vs. 1.58 ± 0.52, *p* = 0.013). The absolute CD3^+^ T‐cell count was numerically lower in virus‐positive patients (746.70 ± 248.23 vs. 829.18 ± 258.67, *p* = 0.108), without reaching statistical significance. No significant difference in CD8^+^ T‐cell counts was observed between the groups (315.42 ± 96.58 vs. 320.76 ± 98.42, *p* = 0.782) (Table 5).

**TABLE 5:** Comparison of peripheral blood T‐lymphocyte subsets between virus‐positive and virus‐negative patients during the first AEB episode.

Variable	Virus‐positive group (*n* = 37)	Virus‐negative group (*n* = 31)	*t*	*p*
CD3^+^ T cells (cells/μL)	746.70 ± 248.23	829.18 ± 258.67	−1.621	0.108
CD4^+^ T cells (cells/μL)	431.28 ± 152.36	508.42 ± 158.94	−2.287	0.025
CD8^+^ T cells (cells/μL)	315.42 ± 96.58	320.76 ± 98.42	−0.277	0.782
CD4^+^/CD8^+^ ratio	1.37 ± 0.48	1.58 ± 0.52	−2.533	0.013

An exploratory ROC analysis of the CD4^+^/CD8^+^ ratio and CD4^+^ T‐cell count is presented in Figure S1, as the predictive performance was limited.

## 5 Discussion

This study of AEB in patients ≥ 70 years revealed several key findings: (1) Respiratory virus detection rate was slightly higher than previously reported in adult bronchiectasis (54.8%), predominantly influenza virus, rhinovirus, and RSV; (2) Virus‐positive patients showed decreased CD4^+^ T cells and reduced CD4^+^/CD8^+^ ratio during AEB; (3) Inflammatory response showed an immunomodulatory pattern characterized by elevated IL‐6 and TNF‐α with relatively low CRP and WBC; (4) CD4^+^/CD8^+^ ratio exhibited moderate discriminatory power for viral differentiation, insufficient for independent clinical decision‐making.

The detection rate of respiratory viruses in this study (54.8%) was slightly higher than the previously reported 25%–49% among adults with AEB [1, 2, 11]. The virus‐positive group showed higher BSI scores than the negative group. This could be due to several factors: (1) In patients with moderate‐to‐severe bronchiectasis, extensive destruction of the bronchial wall and disruption of the mucosal barrier may facilitate viral entry into the lower respiratory tract; (2) Airway mucociliary clearance is more compromised, failing to effectively remove inhaled viruses; (3) Patients with higher BSI scores tend to be older, more malnourished, and have more comorbidities, all contributing to weaker overall immunity; (4) Extensive use of corticosteroid therapy might suppress local interferon and mucosal immunity, weakening antiviral defense.

Influenza virus and RSV were the leading etiological agents, followed by rhinovirus and human metapneumovirus. This distribution pattern is largely consistent with recent multicenter surveillance and retrospective studies [12, 13, 14]. Notably, we identified 6 cases of dual viral infections, accounting for 11.8% of virus‐positive episodes, with influenza virus plus rhinovirus being the most common combination. This rate was higher than that observed in the general population, suggesting that elderly patients' compromised immune function predisposes them to mixed infections [15, 16]. Dual infections typically result in more severe clinical manifestations and prolonged disease course; however, due to limited sample size, we could not further analyze clinical differences between dual and single infections. Additionally, only 3 patients had the same pathogen detected across different AEB episodes, indicating that virus‐associated AEB are predominantly sporadic rather than driven by persistent infection with the same pathogen.

In this study, no significant differences were observed between virus‐positive and virus‐negative groups regarding quality of life scores, pulmonary function parameters, or oxygenation indices. This finding aligns with Gao et al.'s results, demonstrating that AEB associated with viral infections alone do not exhibit more severe respiratory symptoms or pulmonary function impairment compared to bacterial infection‐related AEB [3]. Similarly, Chen et al. reported that bacterial‐viral co‐detection did not result in more severe clinical manifestations as observed in chronic obstructive pulmonary disease [17]. However, this conclusion requires cautious interpretation, as previous study results have been inconsistent [18, 19]. Overall, the differences in clinical severity between virus‐related and non‐virus‐related AEB remain inconsistent, and relying solely on clinical symptoms and routine pulmonary function tests is inadequate for accurately distinguishing viral from bacterial infections.

We observed decreased CD4^+^ T cell counts and reduced CD4^+^/CD8^+^ ratios in virus‐positive patients during AEB. This phenomenon may be mediated by several mechanisms: First, respiratory viruses such as influenza and RSV can directly induce CD4^+^ T cell apoptosis through activation of caspase pathways [20]. Second, virus‐induced cytokine storms (evidenced by elevated IL‐6 and TNF‐α levels observed in this study) may suppress CD4^+^ T cell differentiation and promote exhaustion via the IL‐6/STAT3 signaling pathway [21, 22]. Third, CD4^+^ T cells are recruited from peripheral circulation to infection sites to participate in antiviral responses, resulting in transient reduction of circulating CD4^+^ T cells [23]. The decreased CD4^+^/CD8^+^ ratio reflects an imbalance between CD4^+^ and CD8^+^ T cells. In this study, CD8^+^ T cell counts showed no significant difference between groups, suggesting that the ratio decline was primarily due to selective reduction of CD4^+^ T cells. This imbalance may be related to differential responses of CD4^+^ and CD8^+^ T cells to viral infections: CD4^+^ T cells, serving as helper cells, are more susceptible to exhaustion and apoptosis during antiviral immunity, while CD8^+^ T cells, as cytotoxic cells, remain relatively stable when clearing virus‐infected cells [24, 25]. In previous studies, decreased CD4^+^/CD8^+^ ratios in elderly patients are typically associated with prognosis. The Swedish OCTO and NONA longitudinal immune studies found that inverted CD4^+^/CD8^+^ ratios (< 1) in the very elderly were significantly associated with increased 2‐year mortality and correlated with persistent cytomegalovirus infection [26]. Garrido et al. analyzed adults over 65 years in 2021 and found that those with lower CD4^+^/CD8^+^ ratios exhibited decreased thymic output function, reduced naïve T cell frequencies, elevated inflammatory markers, and trends toward more comorbidities and declining independence in activities of daily living [27].

Although our study did not include follow‐up sampling after clinical recovery, available evidence suggests that the decline in peripheral CD4^+^ T‐cell counts during virus‐associated AEB is at least partially reversible once the acute inflammatory response resolves and lymphocyte redistribution normalizes [28]. However, in elderly individuals, age‐related immunosenescence—including reduced thymic output, impaired naïve T‐cell regeneration, chronic antigenic stimulation, and persistent low‐grade inflammation—may limit the extent of recovery [29]. Notably, CD4^+^ T‐cell counts in both groups in our study were already below the normal adult reference range, indicating a baseline immune deficit in this elderly bronchiectasis population [30]. Therefore, while some improvement in T‐cell subsets may occur after recovery, a full return to “baseline” levels comparable to younger adults is unlikely, and residual immune impairment may persist following AEB.

The advanced age of the study population (≥ 70 years) likely played a central role in shaping the observed clinical, virological, and immunological patterns. Aging is characterized by immunosenescence, including thymic involution, reduced naïve T‐cell output, impaired CD4^+^ T‐cell function, and diminished antiviral immunity, which may explain the lower baseline CD4^+^ T‐cell counts and reduced CD4^+^/CD8^+^ ratios observed in both groups [30]. “Inflammaging,” the chronic pro‐inflammatory state associated with aging, may contribute to the exaggerated IL‐6 and TNF‐α responses in virus‐positive patients [24, 31]. In addition, chronic airway inflammation, recurrent infections, and cumulative inhaled corticosteroid exposure—reported in 61.7% of patients in this study—may further promote T‐cell activation, exhaustion, and impaired recovery of T‐cell subsets [32, 33]. Elderly individuals also exhibit impaired mucociliary clearance, structural airway remodeling, and reduced cough strength, predisposing them to both viral invasion and secondary bacterial infections, consistent with the higher bacterial culture positivity seen in the virus‐positive group. Collectively, these age‐related changes likely contribute to the unique inflammatory profile, immune vulnerability, and infection susceptibility identified in this elderly bronchiectasis cohort.

This study evaluated the value of T lymphocyte subsets in predicting viral infection through ROC curve analysis, revealing that the CD4^+^/CD8^+^ ratio demonstrated moderate discriminatory capacity. Although the predictive performance was inferior to viral nucleic acid detection, T cell testing offers advantages including mature methodology, simple operation, low cost, and high reproducibility, thus retaining clinical significance.

This study has several limitations. First, although it was a prospective cohort study, the sample size was relatively small and derived from a single center, which may limit the generalizability of the findings. Additionally, the study lacks external validation and comprehensive evaluation of other immune markers, and incomplete viral load data prevented analysis of dose–response relationships. Finally, the absence of viral testing during stable periods limits our ability to distinguish pathogenic infections from asymptomatic viral colonization.

## 6 Conclusions

Viral infections are frequent in elderly bronchiectasis patients with AEB and are characterized by reduced CD4^+^ T‐cell counts, lower CD4^+^/CD8^+^ ratios, and heightened inflammatory responses, reflecting underlying age‐related immune vulnerability.

## Author Contributions

**Guoping Zhang:** conceptualization, methodology, formal analysis, writing – original draft. **Yuan Zhuang:** data curation, investigation, formal analysis. **Sai Wang:** investigation, data curation, validation. **Qun Wang:** investigation, resources, data curation. **Jun Qin:** formal analysis, visualization, writing – review and editing. **Zhi Yuan:** resources, supervision, project administration. **Jing Zheng:** conceptualization, supervision, writing – review and editing, funding acquisition, corresponding author.

## Funding

This work was supported by the Hospital‐level Scientific Research Project of the People's Hospital of Fenghua Ningbo (2024KY001).

## Ethics Statement

This study was approved by the Ethics Committee of the People's Hospital of Fenghua Ningbo (Approval No. 2024‐47‐K).

## Conflicts of Interest

The authors declare no conflicts of interest.

## Supporting information


**Figure S1:** ROC Curve Analysis of T‐Lymphocyte Subsets for Predicting Viral Infection ROC analysis was performed to evaluate the predictive performance of the CD4^+^/CD8^+^ ratio and CD4^+^ T‐cell count for viral infection. The CD4^+^/CD8^+^ ratio showed an AUC of 0.708 (95% CI, 0.594–0.821; *p* < 0.05) in predicting viral infection. The optimal cutoff value was 1.54, yielding a sensitivity of 72.1% and a specificity of 70.7%. The CD4^+^ T‐cell count showed an AUC of 0.623 (95% CI, 0.513–0.733; *p* < 0.05). The optimal cutoff value was 415 cells/μL, with a sensitivity of 59.0% and a specificity of 65.9%. Both parameters demonstrated discriminatory capacity for viral infection, with the CD4+/CD8^+^ ratio showing slightly better discriminative performance than the CD4^+^ T‐cell count (Supplementary Figure 1).

## Data Availability

Data will be made available on request.
