# Supplementary material for: Association Between Respiratory Viral Infection and Peripheral T Lymphocyte Subsets in Elderly Patients With Acute Exacerbation of Bronchiectasis
Source: Clin Respir J. 2025 Dec 25;19(12):e70151. doi: 10.1111/crj.70151 (PMC12740622; doi:10.1111/crj.70151)
Supplement: Supplementary file 1 — Figure S1: ROC Curve Analysis of T‐Lymphocyte Subsets for Predicting Viral Infection ROC analysis was performed to evaluate the predictive performance of the CD4+/CD8+ ratio and CD4+ T‐cell count for viral infection. The CD4+/CD8+ ratio showed an AUC of 0.708 (95% CI, 0.594–0.821; p < 0.05) in predicting viral infection. The optimal cutoff value was 1.54, yielding a sensitivity of 72.1% and a specificity of 70.7%. The CD4+ T‐cell count showed an AUC of 0.623 (95% CI, 0.513–0.733; p < 0.05). The optimal cutoff value was 415 cells/μL, with a sensitivity of 59.0% and a specificity of 65.9%. Both parameters demonstrated discriminatory capacity for viral infection, with the CD4+/CD8+ ratio showing slightly better discriminative performance than the CD4+ T‐cell count (Supplementary Figure 1). [file CRJ-19-e70151-s001.docx]

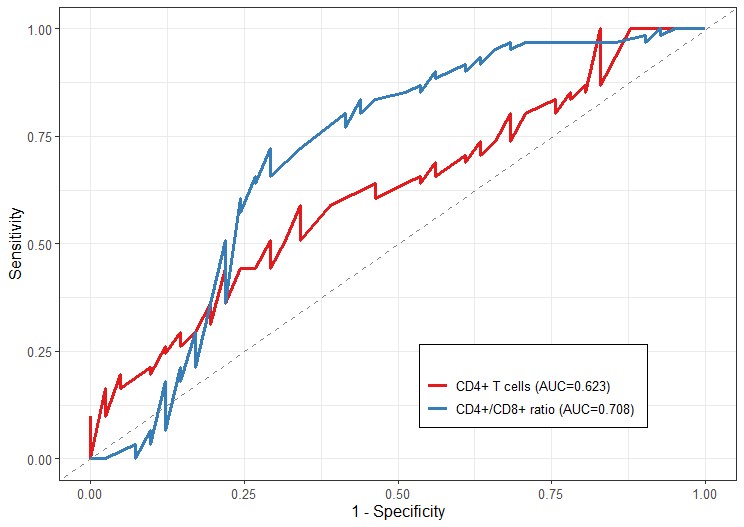


**Supplementary figure 1**

**ROC Curve Analysis of T-Lymphocyte Subsets for Predicting Viral Infection**

ROC analysis was performed to evaluate the predictive performance of the CD4+/CD8+ ratio and CD4+ T-cell count for viral infection. The CD4+/CD8+ ratio showed an AUC of 0.708 (95% CI, 0.594–0.821; P<0.05) in predicting viral infection. The optimal cutoff value was 1.54, yielding a sensitivity of 72.1% and a specificity of 70.7%. The CD4+ T-cell count showed an AUC of 0.623 (95% CI, 0.513–0.733; P<0.05). The optimal cutoff value was 415 cells/μL, with a sensitivity of 59.0% and a specificity of 65.9%. Both parameters demonstrated discriminatory capacity for viral infection, with the CD4+/CD8+ ratio showing slightly better discriminative performance than the CD4+ T-cell count (Supplementary Figure 1).
